# The use of topical anesthetic for a biopsy of the penis

**DOI:** 10.1016/j.jdin.2022.02.003

**Published:** 2022-02-28

**Authors:** Austin Dunn, Tyler Long, John Zampella

**Affiliations:** a Center for Clinical and Cosmetic Research, Aventura, Florida; b Department of Dermatology, Edward Via College of Osteopathic Medicine/LewisGale – Montgomery Dermatology, Blacksburg, Virginia; c Department of Dermatology, New York University Grossman School of Medicine, New York, New York

**Keywords:** biopsy, general dermatology, medical dermatology, oncology, surgery

## Clinical challenge

Skin biopsies of the penis are sometimes necessary to diagnose neoplasms and/or inflammatory eruptions in the groin. Little is published on proper technique, education, and/or comfort with performing genital skin biopsies, and it is an important skill for dermatologists to develop.^1^ Although many components of a penile skin biopsy are similar to those performed on other parts of the body, owing to the sensitivity of the skin in this area, there is often an unusual level of anxiety-provocation in patients undergoing this procedure. To this end, implementing strategies to decrease pain can help make this procedure less stressful for patients and providers alike.

## Solution

To mitigate pain and alleviate anxiety surrounding a genital skin biopsy, the authors have found that the addition of topical anesthetic to the area requiring sampling for 30 minutes before the injection of intradermal lidocaine can greatly decrease the pain from both the needle stick and the burning associated with the injectable anesthetic. The epidermis of the penile skin is approximately 20 μm that allows for rapid penetration of topical medications.^2^ Topical anesthetics are often readily available in most dermatology clinics and/or practices. The implementation of this practice may add several minutes to the duration of the procedure; however, in the authors’ experience, using topical anesthetic leads to demonstrable reductions in patient anxiety levels and improved patient experience.

## Conflicts of interest

None disclosed.
